# Probing the diagnostic values of plasma cf-nDNA and cf-mtDNA for Parkinson’s disease and multiple system atrophy

**DOI:** 10.3389/fnins.2024.1488820

**Published:** 2024-12-02

**Authors:** Chao Ying, Yuan Li, Hui Zhang, Shimin Pang, Shuwen Hao, Songnian Hu, Lifang Zhao

**Affiliations:** ^1^Department of Neurobiology, Xuanwu Hospital, Capital Medical University, Beijing, China; ^2^Beijing Municipal Geriatric Medical Research Center, Beijing, China; ^3^Key Laboratory for Neurodegenerative Diseases of the Ministry of Education, Beijing Key Laboratory on Parkinson’s Disease, Parkinson’s Disease Center for Beijing Institute on Brain Disorders, Clinical and Research Center for Parkinson’s Disease, Capital Medical University, Beijing, China; ^4^National Clinical Research Center for Geriatric Disorders, Xuanwu Hospital, Capital Medical University, Beijing, China; ^5^Department of Neurology, Xuanwu Hospital, Capital Medical University, Beijing, China; ^6^Department of Neurology, The First Hospital of Hebei Medical University, Shijiazhuang, China; ^7^Department of Clinical Biobank and Central Laboratory, Xuanwu Hospital, Capital Medical University, Beijing, China

**Keywords:** circulating cell-free DNA, Parkinson’s disease, multiple system atrophy, biomarker, diagnosis, cell-free nuclear DNA, cell-free mitochondrial DNA

## Abstract

**Background:**

Cell loss and mitochondrial dysfunction are key pathological features of idiopathic Parkinson’s disease (PD) and multiple system atrophy (MSA). It remains unclear whether disease-specific changes in plasma circulating cell-free nuclear DNA (cf-nDNA) and mitochondrial DNA (cf-mtDNA) occur in patients with PD and MSA. In this study, we investigated whether plasma cf-nDNA, cf-mtDNA levels, as well as cf-mtDNA integrity, are altered in patients with PD and MSA.

**Methods:**

TaqMan probe-based quantitative PCR was employed to measure plasma cf-nDNA levels, cf-mtDNA copy numbers, and cf-mtDNA deletion levels in 171 participants, including 76 normal controls (NC), 62 PD patients, and 33 MSA patients. A generalized linear model was constructed to analyze differences in circulating cell-free DNA (cfDNA) biomarkers across clinical groups, while a logistic regression model was applied to assess the predictive values of these biomarkers for developing PD or MSA. Spearman correlations were used to explore associations between the three cfDNA biomarkers, demographic data, and clinical scales.

**Results:**

No significant differences in plasma cf-nDNA levels, cf-mtDNA copy numbers, or cf-mtDNA deletion levels were observed among the PD, MSA, and NC groups (all *P* > 0.05). Additionally, these measures were not associated with the risk of developing PD or MSA. In PD patients, cf-nDNA levels were positively correlated with Hamilton Anxiety Rating Scale scores (Rho = 0.382, FDR adjusted *P* = 0.027). In MSA patients, cf-nDNA levels were positively correlated with International Cooperative Ataxia Rating Scale scores (Rho = 0.588, FDR adjusted *P* = 0.011) and negatively correlated with Montreal Cognitive Assessment scores (Rho = −0.484, FDR adjusted *P* = 0.044). Subgroup analysis showed that PD patients with constipation had significantly lower plasma cf-mtDNA copy numbers than those without constipation (*P* = 0.049). MSA patients with cognitive impairment had significantly higher cf-nDNA levels compared to those without (*P* = 0.008).

**Conclusion:**

Plasma cf-nDNA level, cf-mtDNA copy number, and cf-mtDNA deletion level have limited roles as diagnostic biomarkers for PD and MSA. However, their correlations with clinical symptoms support the hypothesis that cell loss and mitochondrial dysfunction are involved in PD and MSA development.

## Introduction

Parkinson’s disease (PD) is a gradually progressive and highly disabling disorder of the central nervous system, primarily affecting the elderly (Opara et al., 2017). The primary clinical manifestations of PD include bradykinesia, rigidity, resting tremor, and postural instability. Non-motor symptoms encompass a reduced sense of smell, depression, and dementia (Calne, 2005; Kummer et al., 2009). Lewy bodies and dopaminergic neuron degeneration are hallmark pathological features of PD. Motor symptoms emerge when 60–80% of dopaminergic neurons in the striatum within the substantia nigra pars compacta are lost (Fearnley and Lees, 1991; Eriksen et al., 2005). Multiple system atrophy (MSA) is a rare and rapidly progressive neurodegenerative disorder classified as an alpha-synucleinopathy. It is characterized by various combinations of clinical manifestations, including autonomic dysfunction, Parkinson’s syndrome, cerebellar ataxia, and pyramidal signs. The neuropathological hallmark of MSA is the accumulation of abnormal fibrillar alpha-synuclein in glial cells, forming glial inclusions (Jellinger and Wenning, 2016). Limited accessibility to the central nervous system, combined with the complexity and heterogeneity of symptoms, makes early and accurate diagnosis of these diseases is challenging (Tolosa et al., 2006; Schrag et al., 2015; Dutta et al., 2023). A minimally invasive and reliable assay for early and precise diagnosis of PD and MSA is urgently needed (Postuma and Berg, 2016).

Mitochondrial dysfunction is closely linked to the pathogenesis of PD and MSA. Mitochondria, the cell’s energy reservoirs, contain their own DNA. Mitochondrial DNA (mtDNA) is a multicopy extrachromosomal genome that is transcribed and replicated independently of the cell cycle and directs mitochondrial protein synthesis. The mitochondrial genome consists of a double-stranded loop with an outer heavy chain and an inner light chain. The coding region contains 13 genes for the electron transport chain complex, 22 transfer RNAs, 2 ribosomal RNAs, and a non-coding control region with the promoter and the initiation site for heavy chain replication (Phillips et al., 2014). Mitochondrial DNA, typically located near the inner mitochondrial membrane, is exposed to high levels of reactive oxygen species and free radicals generated by the electron transport chain, rendering it susceptible to damage (Raha and Robinson, 2000; Masayesva et al., 2006). Mitochondrial dysfunction is a recognized mechanism potentially involved in the pathogenesis of synucleinopathies, such as PD and MSA. Mitochondrial genome integrity defects have been observed in idiopathic preclinical models and postmortem PD brain tissue (Sanders et al., 2014; Gonzalez-Hunt and Sanders, 2021). Dysfunction in mitochondrial respiratory chain activity, particularly in complex I, has been observed in PD (Schapira et al., 1989). Mitochondrial inhibitors like MPTP and fisetin one contribute to the clinical and neuropathological features of PD (Hatcher et al., 2008). Mitochondrial DNA alterations, including copy number reductions, deletions, point mutations, and impaired maintenance, have been associated with PD development (Autere et al., 2004; Pyle et al., 2016). While most studies focus on PD, mitochondrial defects have also been identified in MSA (Compagnoni et al., 2018). Two studies evaluated respiratory chain activity in MSA patients and healthy controls found reduced complex I activity in patients’ skeletal muscle (Blin et al., 1994), but not in platelets or substantia nigra (Gu et al., 1997). Furthermore, studies have reported selective reductions in coenzyme Q10 levels in the cerebellar regions of MSA patients and decreased plasma levels of coenzyme Q10 in these patients as well (Barca et al., 2016; Mitsui et al., 2016; Schottlaender et al., 2016).

Circulating cell-free DNA (cfDNA) carries markers indicative of mitochondrial dysfunction. cfDNA typically comprises nuclear and mitochondrial DNA fragments in the bloodstream, mainly 160–180 bp in length, derived from apoptosis and necrosis (Bruno et al., 2020). The short half-life of cfDNA, approximately 2 h, enables it to accurately reflect dynamic changes in disease-related pathological conditions (Rostami et al., 2020; Loft et al., 2023). cfDNA can be detected months before radiological changes and is used for multiple non-invasive longitudinal assessments. cfDNA can cross the blood-brain barrier, significantly enhancing its clinical utility compared to conventional biomarkers (Loft et al., 2023). Several studies have explored the role of cfDNA in diagnosing and understanding neurodegenerative diseases like PD, with a primary focus on concentration quantification and methylation analysis (Gaitsch et al., 2023; Malhotra et al., 2023). Wojtkowska et al. (2024) analyzed serum circulating cell-free nuclear DNA (cf-nDNA) levels in 30 PD patients and 15 controls by measuring the expression of the nuclear gene *KRAS*, revealing a slight but significant increase in PD patients. However, the increase of cf-nDNA level in PD patients disappeared after adjusting for age (Wojtkowska et al., 2024). In contrast, Scalzo et al. (2009) reported significantly lower cf-nDNA levels in PD patients compared to controls, based on the expression of the nuclear gene β*-globin* in 42 PD patients and 20 controls. Additionally, some studies analyzed circulating cell-free mitochondrial DNA (cf-mtDNA) levels in cerebrospinal fluid (CSF) and serum of PD patients, though the results were inconsistent (Müller-Nedebock et al., 2019; Kunze et al., 2023). Another critical issue is that current cf-mtDNA studies primarily rely on cerebrospinal fluid (CSF) and serum. Due to the invasive nature of CSF collection and the risk of leukocyte DNA contamination in serum, plasma-derived cfDNA may be more suitable for biomarker studies (Kerachian et al., 2021; Malhotra et al., 2023).

This study aimed to evaluate whether plasma cf-nDNA level, cf-mtDNA copy number, and cf-mtDNA deletion level are altered in patients with PD and MSA, and whether these changes correlate with the risk of developing PD and MSA. Furthermore, we explored the relationships between these cfDNA markers and motor as well as non-motor symptoms in patients with PD and MSA.

## Materials and methods

### Study subjects

This cross-sectional study recruited 171 participants from May to November 2022 at Xuanwu Hospital, Capital Medical University. Sixty-two participants with PD were diagnosed using the 2015 MDS clinical diagnostic criteria (Postuma et al., 2015). Individuals with a first- or second-degree relative with a family history of PD or onset before age 50 were excluded. Probable MSA was diagnosed according to the second consensus statement on MSA diagnosis (Gilman et al., 2008). Patients were then classified into two subtypes based on predominant motor symptoms: MSA-P (predominant parkinsonism) and MSA-C (predominant cerebellar ataxia). Diagnoses were confirmed by at least two experienced specialists in movement disorders. Patients were excluded if they had: (i) Severe dementia or communication difficulties; (ii) Complications like aphasia and severe dysarthria affecting clinical evaluation; (iii) Parkinsonian syndromes due to cerebrovascular, hypoxic, traumatic, infectious, metabolic, or systemic diseases affecting the central nervous system (Li et al., 2020). The study included 76 NCs with no known medical conditions. Exclusion criteria for NCs included a family history of synucleinopathy, neuropathy, psychiatric illness, a history of head injury, or signs of Rapid Eye Movement sleep behavior disorder (RBD). Additionally, individuals with cancer, end-stage renal disease, active infections, significant trauma, autoimmune disease, or chronic inflammatory disorders were excluded from both groups. All procedures were approved by the Xuanwu Hospital Medical Research Ethics Committee and Institutional Review Board (approval number: [2022]047), following the Declaration of Helsinki. All participants and/or their legal proxies provided written informed consent.

### Clinical assessment

Site investigators thoroughly assessed participants’ demographics and clinical characteristics. Blood samples were collected within one week of the clinical assessments. Demographic data included age, sex, body mass index (BMI), education level, disease duration, and smoking and drinking history. BMI was calculated as weight (kg) divided by height squared (m^2^). Smoking and drinking history were categorized as “ever” or “never.” Disease duration was defined as the time from the onset of first motor symptoms to the blood sampling date. Medical histories of diabetes, hypertension, coronary heart disease, cerebrovascular disease, and hyperlipidemia were self-reported. Clinical characteristics were assessed using multiple scales for motor and non-motor symptoms. Motor symptoms were assessed using part III of the Movement Disorder Society-Unified Parkinson’s Disease Rating Scale (MDS-UPDRS) for PD and MSA patients, and the Unified Multiple System Atrophy Rating Scale (UMSARS) for MSA patients. PD patients with Hoehn and Yahr (H&Y) stage ≤ 2 were considered to have early-stage PD, while those with H&Y stage 2.5–5 were classified as intermediate- to late-stage PD. Cognitive function was assessed using the Mini-Mental State Examination (MMSE) and Montreal Cognitive Assessment (MoCA). Depression and anxiety were evaluated using the Hamilton Depression Rating Scale (HAM-D) and Hamilton Anxiety Rating Scale (HAM-A). RBD symptom history and severity were evaluated using the RBD Questionnaire-Hong Kong (RBDQ-HK). The overall non-motor symptoms burden was assessed using the Non-Motor Symptom Scale (NMSS), and constipation was screened by the part 21 of NMSS. Olfactory function was assessed using the Argentine Hyposmia Rating Scale (AHRS). The International Cooperative Ataxia Rating Scale (ICARS) was used to evaluate ataxia severity in MSA patients. PD Motor subtypes were classified as tremor dominant (TD), postural instability and gait difficulty (PIGD), or mixed (MIX) phenotype based on the ratio of mean tremor score to mean PIGD score in MDS-UPDRS (Stebbins et al., 2013). Considering the effect of education level on cognition, 1 point was added to MoCA scores (< 30) for subjects with ≤ 12 years of education. H&Y stage and MDS-UPDRS were assessed in the OFF state for PD patients.

### Sample collection and cfDNA isolation

Blood samples were collected in EDTA tubes and centrifuged at 1,600 *g* for 10 min at 4°C, within 2 h of collection. The supernatant, free of cellular components, was carefully transferred to a new tube, minimizing disruption of the cell pellet. A second centrifugation at 16,000 *g* for 10 min at 4°C was performed to remove residual cellular debris. The clarified supernatant was gently mixed by inversion to achieve uniformity aliquoted, and stored at −80°C for cfDNA extraction. Plasma stored at −80°C was purified for cfDNA within six months. cfDNA was isolated from 500 μL of plasma aliquots using the VAHTS^§^ Serum/Plasma Circulating DNA Kit (Vazyme Biotech, Nanjing, China) according to the manufacturer’s protocol. The eluted cfDNA was divided into three aliquots to minimize degradation from repeated freeze-thaw cycles and stored at −80°C.

### Quantification of cf-nDNA levels, cf-mtDNA copy numbers and cf-mtDNA deletion levels

TaqMan-based quantification of cf-nDNA and cf-mtDNA levels was performed using established methods (Lowes et al., 2019; Davis et al., 2020). The nuclear housekeeping gene (*RPP30*) and two mitochondrial regions (*MT-ND1* and *MT-ND4*) were measured in three independent PCR runs and were expressed as ng/mL of plasma. *RPP30* levels represent total plasma cf-nDNA concentration. cf-mtDNA copy number was determined by comparing mitochondrial *MT-ND1* to the single-copy nuclear gene *RPP30*. cf-mtDNA deletion level was expressed as the ratio of *MT-ND1* to *MT-ND4*. *RPP30* was amplified and quantified using the following primers and probe: 5′-AGATTTGGACCTGCGAGCG-3′ (forward), 5′-GAGCGGCTGTCTCCACAAGT-3′ (reverse), 5′-FAM-TTCTGACCTGAAGGCTCTGCGCG-BHQ1-3′ (probe). The *ND1* region was amplified and quantified using the following primers and probe: 5′-CCCTAAAACCCGCCACATCT-3′ (forward), 5′-GAGCGATGGTGAGAGCTAAGGT-3′ (reverse), 5′-HEX-CCATCACCCTCTACATCACCGCCC-BHQ1-3′ (probe). The *ND4* region was amplified and quantified using the following primers and probe: 5′-CCATTCTCCTCCTATCCCTCAAC-3′ (forward), 5′-CACAATCTGATGTTTTGGTTAAACTATATTT-3′ (reverse), 5′-FAM-CCGACATCATTACCGGGTTTTCCTCTTG-BHQ1-3′ (probe). Each reaction was run on a LightCycler 480 (Roche, Mannheim, Germany) in a 20 μL volume, containing 2 μL of isolated cfDNA template, 2 μL of 10 × Ex Taq Buffer (Mg^2+^ plus) (20 mM), 1 μL of dNTP Mixture (2.5 mM each), 0.2 μL of TaKaRa Ex Taq HS (5 U/μL), 0.4 μL of each primer and probe (10 μM), and 13.6 μL of double-distilled water. Thermal cycling profiles for *RPP30*, *ND1*, and *ND4* included initial denaturation at 94°C for 1 min, followed by 40 cycles of denaturation at 95°C for 15 s, and annealing/extension at 64°C for 1 min, with fluorescence data collected at 64°C.

### Quality control of qPCR

To ensure accuracy and reliability, blinded quantitative polymerase chain reaction (qPCR) assays were performed following a standardized and rigorous approach. Melting curve analysis was conducted after each reaction to confirm PCR product specificity and identity. Triplicate reactions were performed, and mean values were calculated with a standard deviation (SD) of less than 0.5 for Cq values. All cfDNA samples were required to fall within the linear range of a standard curve established through serial dilutions of pooled human leukocyte genomic DNA. Amplified target concentrations were computed by correlating Cq values with the standard curve, accounting for dilution factors from the initial plasma sample to the final PCR reaction. The standard curve concentration range was 4.0 ng/μL to 0.0165 ng/μL for *RPP30*, and 38.0 ng/μL to 0.125 ng/μL for *ND1* and *ND4*. Standard curve accuracy was confirmed by ensuring an *R*^2^ value greater than 0.999 and amplification efficiency between 90 – 110%. Calibrator DNA from healthy donors was included on each plate to normalize Cq values and compensate for inter-plate variations in PCR efficiency. The inter-assay coefficient of variation was calculated for each target, with measurements repeated if it exceeded two SD. A negative control was always included to ensure no product detection until at least five cycles beyond the lowest concentration on the standard curve, avoiding false-positive results.

### Statistical analysis

Sample size was not determined using statistical methods but was similar to that in previous studies (Park et al., 2022; Wojtkowska et al., 2024). Participants were classified into NC, PD, and MSA groups by diagnoses. Baseline characteristics were summarized and compared between groups. Continuous variables were evaluated for normality with the Shapiro–Wilk test and homogeneity with Levene’s chi-square test. Normally distributed data were reported as mean ± SD and compared with one-way ANOVA. Non-normally distributed data were presented as median with interquartile range and compared using the Mann–Whitney U or Kruskal–Wallis H test. Categorical variables were presented as counts (percentage) and compared with chi-square tests or Fisher’s exact test. Additional *post hoc* comparisons were conducted between groups using Bonferroni correction. A general linear regression model was used to compare plasma cf-nDNA levels, cf-mtDNA copy numbers, and cf-mtDNA deletion levels across disease groups and subgroups, adjusting for age, sex, BMI, and education level. Spearman’s rank correlation analyses with false discovery rate (FDR) adjustment were performed, controlling for age, sex, BMI, and education level, to assess correlations between cfDNA biomarkers and various clinical factors, including age, disease duration, H&Y stage, MMSE, MoCA, HAM-D, HAM-A, RBDQ-HK, UPDRS-III, NMSS, UMSARS, and ICARS scores. Univariate and multivariate logistic regression models were constructed to compute odds ratio (OR) and 95% confidence interval (CI) to evaluate the association between three significant biomarkers and the risk of PD and MSA. The multivariate model was adjusted for age, sex, BMI, education level, and other potential confounders. Additionally, cfDNA biomarkers were included in models as continuous variables (per SD) and categorical variables (in tertiles) to verify the robustness of the association. Linear trend was examined by using the median of each tertile subgroup as a continuous variable. All statistical analyses were conducted using SPSS V.22.0 (IBM Corp., New York, NY, USA) and R3.2.3 (AT&T, now Lucent Technologies, Vienna, Austria). A two-tailed *P* < 0.05 was considered statistically significant.

## Results

### Subject characteristics

The study included 171 participants: 62 with PD, 33 with MSA, and 76 NC. Table 1 presents the baseline demographic and clinical characteristics of participants. Significant differences in age and education levels were observed among the NC, MSA, and PD groups. Additionally, distinct differences in lifestyle factors (e.g., smoking) and comorbid conditions (e.g., hypertension, coronary heart disease, cerebrovascular disease) were observed among the three groups. Participants with PD and MSA scored significantly lower on cognitive assessments, as indicated by lower MMSE and MoCA scores. Furthermore, the PD and MSA groups had significantly higher scores on the HAM-D, the HAM-A, and the RBDQ-HK compared to the NC group. No significant differences in the prevalence of hyperlipidemia were observed among the three groups.

**TABLE 1:** Baseline characteristics of the participants in the study.

Characteristics	NC (*n* = 76)	PD (*n* = 62)	MSA (*n* = 33)
Age, years	70.05 ± 5.12	65.77 ± 5.63***^‡‡^	62.39 ± 4.23^†††^
Female (%)	50 (65.8%)	40 (64.5%)	15 (45.5%)
BMI	23.95 [22.00; 25.83]	24.35 [22.75; 25.98]	25.10 [23.80; 26.40]
Education, years	12.58 [11.00; 15.00]	11.43 [9.00; 15.00]	10.94 [9.00; 12.00]^†^
Disease duration, years	NA	3.03 [1.80; 4.64]	2.37 [1.89; 3.27]
Smoke (%)	6 (7.9%)	12 (19.4%)	12 (36.4%)^†††^
Drink (%)	30 (39.5%)	14 (22.6%)	9 (27.3%)
Hypertension (%)	31 (40.8%)	30 (48.4%)^‡^	7 (21.2%)
Diabetes (%)	11 (14.5%)	15 (24.2%)	10 (30.3%)
Coronary heart disease (%)	2 (2.6%)	13 (21.0%)**	1 (3.0%)
Cerebrovascular disease (%)	0 (0.0%)	15 (24.2%)***	6 (18.2%)^††^
Hyperlipidemia (%)	29 (38.2%)	15 (24.2%)	12 (36.4%)
H&Y stage	NA	2.00 [2.00; 2.00]^‡‡‡^	2.50 [2.00; 3.00]
MDS-UPDRS-III	NA	32.00 [22.00; 42.00]	29.00 [21.00; 39.00]
UMSARS-II	NA	NA	15.38 ± 7.02
ICARS scores	NA	NA	21.39 ± 9.93
NMSS scores	NA	29.00 [8.00; 55.00]	37.00 [26.00; 61.00]
AHRS scores	NA	20.00 [12.00; 24.00]^‡‡‡^	24.00 [24.00; 24.00]
MMSE scores	29.00 [28.00; 30.00]	27.00 [25.25; 28.00]***	27.00 [26.00; 28.00]^†††^
MOCA scores	26.00 [25.00; 27.25]	22.50 [20.00; 26.00]***	22.00 [20.00; 25.00]^†††^
HAM-D scores	2.00 [1.00; 4.00]	7.00 [2.00; 13.00]***	6.00 [3.00; 13.00]^†††^
HAM-A scores	4.50 [2.00; 7.25]	9.50 [3.25; 17.75]***	10.00 [4.00; 17.00]^††^
RBDQ-HK scores	1.00 [0.00; 3.00]	15.00 [2.00; 32.75]***	32.00 [22.00; 49.00]^†††^
cf-nDNA level (ng/ml)	8.66 [6.20; 12.38]	8.51 [6.43; 9.95]	7.48 [5.84; 9.30]
ND1 (ng/ml)	95.69 [48.55; 137.23]	73.88 [33.22; 133.23]	73.01[49.94; 127.62]
ND4 (ng/ml)	86.46 [44.37; 129.09]	69.46 [30.62; 123.68]	67.46 [44.08; 119.32]
cf-mtDNA copy number	10.26 [4.91; 17.18]	9.39 [3.91; 16.78]	9.19 [6.65; 16.68]
cf-mtDNA deletion level	1.07 ± 0.07	1.07 ± 0.06	1.08 ± 0.07

Continuous variables are reported as mean ± standard deviation or median (interquartile range), and categorical variables are displayed as numbers (%). BMI, body mass index; NC, normal control; H&Y, Hoehn and Yahr; MDS-UPDRS-III: movement disorder society-unified Parkinson’s disease rating scale part III; UMSARS, unified multiple system atrophy rating scale part II; NMSS, non-motor symptom scale; MMSE, mini-mental state examination; AHRS, Argentine Hyposmia Rating Scale; ICARS, international cooperative ataxia rating scale; MoCA, montreal cognitive assessment; HAM-D, Hamilton depression scale; HAM-A, Hamilton anxiety scale; RBDQ-HK, rapid eye movement sleep behavior disorder questionnaire-Hong Kong; PD, Parkinson’s disease; MSA, multiple system atrophy; cf-mtDNA, circulating cell-free mitochondrial DNA; cf-nDNA, circulating cell-free nuclear DNA; NA, not applicable.

PD vs. NC, **P < 0.01,

***P < 0.001; MSA vs. NC,

^†^*P* < 0.05,

^††^*P* < 0.01,

^†††^*P* < 0.001;

PD vs. MSA, ^‡^*P* < 0.05,

^‡‡^*P* < 0.01,

^‡‡‡^*P* < 0.001.

### Intergroup and subgroup comparisons of plasma cfDNA biomarkers

**FIGURE 1:**
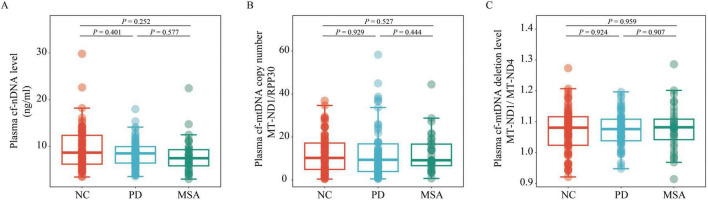
Comparison of plasma cf-nDNA levels, cf-mtDNA copy numbers and deletion levels among NC (*n* = 76), PD (*n* = 62), and MSA (*n* = 33). The figure illustrates plasma: **(A)** cf-nDNA level, represented by *RPP30*; **(B)** cf-mtDNA copy number, represented by the ratio of *MT-ND1* to *RPP30*; **(C)** cf-mtDNA deletion level, represented by the ratio of *MT-ND1* to *MT-ND4*, across the three groups. Statistical analysis was performed using a generalized linear model, adjusting for age, gender, BMI, and educational level. Boxplots indicate the median and IQR. The upper whisker extends to the highest value within 1.5 times the IQR, and the lower whisker to the lowest value within 1.5 times the IQR. Data points outside the whiskers are classified as “outliers.” PD, Parkinson’s disease; MSA, multiple system atrophy; NC, normal control; cf-mtDNA, circulating cell-free mitochondrial DNA; cf-nDNA, circulating cell-free nuclear DNA; IQR, interquartile range.

**FIGURE 2:**
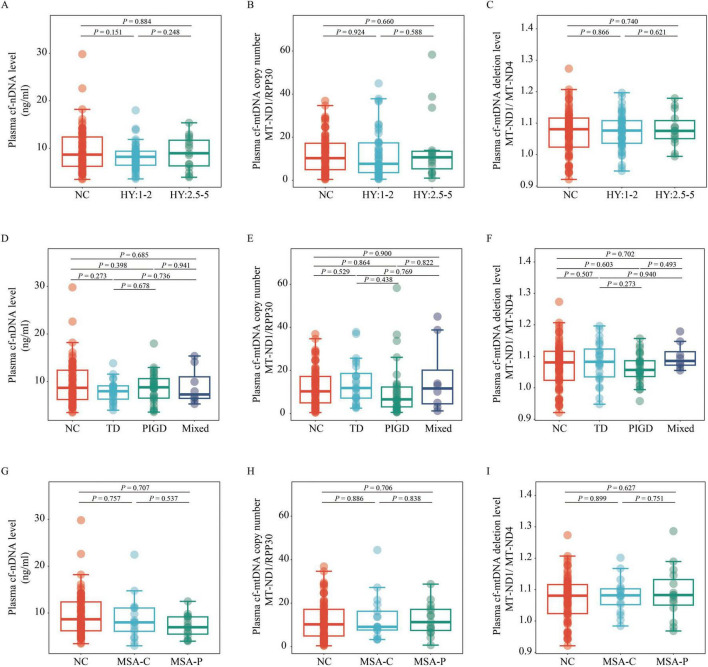
Comparison of plasma cf-nDNA levels, cf-mtDNA copy numbers and cf-mtDNA deletion levels between NC (*n* = 76) and patients with various PD and MSA subtypes. The figure displays variations in plasma cf-nDNA levels [*RPP30*; panels **(A,D,G)**], cf-mtDNA copy numbers [*MT-ND1* to *RPP30* ratio; panels **(B,E,H)]**, and cf-mtDNA deletion levels [*MT-ND1* to *MT-ND4* ratio; panels **(C,F,I)**]. Panels **(A,B,C)** classify PD into early stages (H&Y stage 1–2; *n* = 47) and mid-late stages (H&Y stage 2.5–5; *n* = 15). Panels **(D,E,F)** classify PD into TD (*n* = 24), PIGD (*n* = 30), and Mix (*n* = 8) groups. Panels **(G,H,I)** classify MSA into MSA-C (*n* = 18) and MSA-P (*n* = 15) subtypes. Statistical analysis was conducted using multivariable linear regression, adjusted for age, sex, BMI, and education level, to assess *P*-values. Boxplots depict the median and IQR, with whiskers extending to values within 1.5 times the IQR. Data points outside the whiskers are labeled as “outliers.” PD, Parkinson’s disease; MSA, multiple system atrophy; NC, normal control; TD, tremor dominant; PIGD, postural instability and gait disorder; Mix, mixed symptomatology; H&Y, Hoehn and Yahr; MSA-C, Multiple System Atrophy-Cerebellar type; MSA-P, Multiple System Atrophy-Parkinsonian type; cf-mtDNA, circulating cell-free mitochondrial DNA; cf-nDNA, circulating cell-free nuclear DNA; IQR, interquartile range.

A generalized linear model, adjusted for age, sex, BMI, and education level, was used to compare cf-nDNA levels, cf-mtDNA copy numbers and cf-mtDNA deletion levels across the different groups. The analysis showed no significant differences in plasma cf-nDNA levels between the PD, MSA, and NC groups (all *P* > 0.05; Figure 1A). Similarly, cf-mtDNA copy numbers and deletion levels did not differ significantly among the PD, MSA, and NC groups (all *P* > 0.05; Figures 1B, C). Additionally, when grouping PD patients according to disease stage and motor typing, or grouping MSA patients according to disease subtype, no significant differences were observed in the three plasma cfDNA biomarkers among these groupings (all *P* > 0.05; Figures 2A–I).

### Associations between cfDNA biomarkers and risk of developing PD and MSA

We investigated the associations between cfDNA biomarkers and the risks of developing PD and MSA. After adjusting for age, sex, BMI, education level, hypertension, coronary heart disease, and cerebrovascular disease, no significant associations were found between cf-nDNA level (OR: 0.74; 95% CI: 0.42–1.20; *P* = 0.247), cf-mtDNA copy number (OR: 1.21; 95% CI: 0.75–1.92; *P* = 0.427), or cf-mtDNA deletion level (OR: 1.02; 95% CI: 0.64–1.61; *P* = 0.943) and the risk of developing PD (Table 2). Similar findings were observed when the levels of the three cfDNA markers were stratified into tertiles. Additionally, trend tests did not show significant linear correlations between cf-nDNA level (*P* for trend = 0.422), cf-mtDNA copy number (*P* for trend = 0.462), or cf-mtDNA deletion levels (*P* for trend = 0.689) and the risk of developing PD (Table 2).

**TABLE 2:** Associations between cfDNA biomarkers and risk of developing MSA and PD in logistic regression models.

Plasma measures	PD						MSA					
	**Crude model**			**Multivariate model**			**Crude model**			**Multivariate model**		
	**OR**	**95% CI**	***P*-value**	**OR**	**95% CI**	***P*-value**	**OR**	**95% CI**	***P*-value**	**OR**	**95% CI**	***P*-value**
**cf-nDNA level**												
Per SD	0.73	0.50–1.05	0.102	0.74	0.42–1.20	0.247	0.65	0.38–1.03	0.094	1.39	0.68–2.74	0.340
Q1	ref			ref			ref			ref		
Q2	1.55	0.68–3.55	0.298	1.56	0.51–4.93	0.437	1.13	0.43–2.95	0.808	2.03	0.40–1.16	0.401
Q3	0.58	0.24–1.34	0.202	0.65	0.20–2.04	0.463	0.34	0.11–1.00	0.058	3.06	0.50–2.34	0.243
*P*-value for trend			0.162			0.422			**0.046**			0.252
**cf-mtDNA copy number**												
Per SD	1.06	0.75–1.49	0.742	1.21	0.75–1.92	0.427	1.04	0.68– 1.56	0.851	0.97	0.50–1.91	0.937
Q1	ref			ref			ref			ref		
Q2	1.19	0.52–2.71	0.676	1.69	0.54–5.48	0.372	1.47	0.54–4.06	0.449	1.15	0.16–7.67	0.885
Q3	0.77	0.33–1.75	0.527	0.76	0.23–2.48	0.651	0.96	0.34–2.72	0.943	1.06	0.18–6.22	0.945
*P*-value for trend			0.458			0.462			0.831			0.987
**cf-mtDNA deletion level**												
Per SD	1.04	0.74–1.47	0.823	1.02	0.64–1.61	0.943	1.11	0.73–1.69	0.619	1.31	0.66–2.69	0.447
Q1	ref			ref			ref			ref		
Q2	1.31	0.57–3.01	0.527	0.98	0.33–2.93	0.975	1.73	0.65–4.79	0.278	1.56	0.30–8.36	0.594
Q3	0.83	0.36–1.93	0.670	0.78	0.24–2.45	0.672	0.87	0.30–2.49	0.789	1.26	0.25–6.51	0.781
*P*-value for trend			0.714			0.689			0.945			0.748

Bold values: *P-*value < 0.05. Crude model: unadjusted model; Multivariate model: adjusted for age, sex, BMI, education level, hypertension, coronary heart disease, and cerebrovascular disease in PD patients; adjusted for age, sex, BMI, education level, smoking status, and cerebrovascular disease in MSA patients; Trend analysis utilized the median value of each tertile. cf-mtDNA, circulating cell-free mitochondrial DNA; cf-nDNA, circulating cell-free nuclear DNA; PD, Parkinson’s disease; MSA, multiple system atrophy; OR, odds ratio; CI, confidence interval; SD, standard deviation; -, not available.

In MSA patients, those in the highest tertile of cf-nDNA level had a 64% lower risk of developing MSA compared to those in the lowest tertile (OR: 0.34; 95% CI: 0.11–1.00; *P* = 0.058), indicating a significant dose-response relationship (*P* for trend = 0.046). However, these associations were no longer significant after adjusting for age, sex, BMI, education level, smoking status, and cerebrovascular disease (OR: 3.06; 95% CI: 0.50–2.34; *P* = 0.243; *P* for trend = 0.252). Similarly, no significant associations were observed between cf-nDNA level (OR: 1.39; 95% CI: 0.68–2.74; *P* = 0.340), cf-mtDNA copy number (OR: 0.97; 95% CI: 0.50–1.91; *P* = 0.937), or cf-mtDNA deletion level (OR: 1.31; 95% CI: 0.66–2.69; *P* = 0.447) per SD and the risk of developing MSA, after adjusting for confounding factors (Table 2).

### Associations between plasma cfDNA biomarkers and clinical features

We performed correlation analyses between plasma cfDNA biomarkers and clinical characteristics in different subgroups, correcting for confounders such as age, sex, years of education, and BMI, with FDR correction. The results showed a significantly positive correlation between plasma cf-nDNA levels and HAM-A scores in PD patients (Rho = 0.382, FDR adjusted *P* = 0.027). Similarly, in MSA patients, cf-nDNA levels showed a significantly positive correlation with ICARS scores (Rho = 0.588, FDR adjusted *P* = 0.011) and a significantly negative correlation with MoCA scores (Rho = −0.484, FDR adjusted *P* = 0.044). No correlations were found for cf-nDNA level, cf-mtDNA copy number and cf-mtDNA deletion level with other clinical characteristics (Table 3).

**TABLE 3:** Spearman correlations between plasma cfDNA biomarkers and clinical parameters across the diagnostic groups.

Group	Plasma measures		Duration	H&Y stage	MMSE	MOCA	HAM-D	HAM-A	RBDQ-HK	MDS-UPDRS-III	NMSS	UMSARS-II	ICARS
NC	cf-nDNA level	Rho			0.227	0.082	0.228	0.208	−0.185				
		*P*			0.055	0.494	0.054	0.080	0.121				
		FDR adjusted *P*			0.133	0.494	0.133	0.133	0.151				
	cf-mtDNA copy number	Rho			0.070	−0.139	0.098	−0.034	0.167				
		*P*			0.559	0.243	0.414	0.775	0.160				
		FDR adjusted *P*			0.699	0.608	0.690	0.775	0.608				
	cf-mtDNA deletion level	Rho			0.033	−0.040	0.214	0.059	0.016				
		*P*			0.786	0.743	0.076	0.625	0.897				
		FDR adjusted *P*			0.897	0.897	0.380	0.897	0.897				
PD	cf-nDNA level	Rho	0.182	0.060	−0.108	−0.123	0.196	0.382	0.107	−0.073	0.184		
		*P*	0.171	0.656	0.422	0.357	0.141	0.003	0.426	0.591	0.172		
		FDR adjusted *P*	0.387	0.656	0.548	0.548	0.387	**0.027**	0.548	0.656	0.387		
	cf-mtDNA copy number	Rho	−0.136	0.013	0.089	0.097	−0.135	−0.191	−0.186	−0.025	−0.057		
		*P*	0.309	0.923	0.504	0.469	0.311	0.150	0.162	0.852	0.675		
		FDR adjusted *P*	0.700	0.923	0.756	0.756	0.700	0.700	0.700	0.923	0.868		
	cf-mtDNA deletion level	Rho	0.114	−0.072	0.052	0.003	−0.025	−0.038	−0.019	−0.149	−0.184		
		*P*	0.397	0.595	0.703	0.981	0.885	0.779	0.886	0.273	0.174		
		FDR adjusted *P*	0.981	0.981	0.981	0.981	0.981	0.981	0.981	0.981	0.981		
MSA	cf-nDNA level	Rho	0.271	0.251	−0.382	−0.484	0.267	0.186	0.048	0.178	0.444	0.412	0.588
		*P*	0.154	0.189	0.041	0.008	0.162	0.334	0.805	0.354	0.016	0.029	0.001
		FDR adjusted *P*	0.255	0.260	0.090	**0.044**	0.255	0.389	0.805	0.389	0.059	0.080	**0.011**
	cf-mtDNA copy number	Rho	−0.051	−0.230	0.080	0.184	0.250	0.145	−0.021	−0.088	−0.108	−0.189	−0.153
		*P*	0.792	0.230	0.681	0.339	0.190	0.452	0.914	0.650	0.578	0.336	0.447
		FDR adjusted *P*	0.871	0.829	0.832	0.829	0.829	0.829	0.914	0.832	0.832	0.829	0.829
	cf-mtDNA deletion level	Rho	0.034	−0.090	−0.289	0.015	0.007	0.035	0.060	0.230	0.026	0.123	0.014
		*P*	0.859	0.641	0.129	0.937	0.970	0.857	0.757	0.231	0.894	0.532	0.946
		FDR adjusted *P*	0.970	0.970	0.970	0.970	0.970	0.970	0.970	0.970	0.970	0.970	0.970

Bold values: FDR adjusted *P*-value < 0.05. Adjusted for age, sex, BMI and education years. NC, normal control; H&Y, Hoehn and Yahr; MDS-UPDRS-III, movement disorder society-unified Parkinson’s disease rating scale part III; UMSARS, unified multiple system atrophy rating scale part II; NMSS, non-motor symptom scale; MMSE, mini-mental state examination; MoCA, montreal cognitive assessment; HAM-D, Hamilton depression scale; HAM-A, Hamilton anxiety scale; RBDQ-HK, rapid eye movement sleep behavior disorder questionnaire-Hong Kong; ICARS, international cooperative ataxia rating scale; cf-mtDNA, circulating cell-free mitochondrial DNA; cf-nDNA, circulating cell-free nuclear DNA; FDR, false discovery rate; PD, Parkinson’s disease; MSA, multiple system atrophy.

We also performed subgroup comparisons of plasma cfDNA biomarkers in PD and MSA patients with different clinical presentations, correcting for confounders such as age, sex, years of education, and BMI (Table 4). We found that plasma cf-mtDNA copy numbers were significantly lower in PD patients with constipation compared to those without constipation (*P* = 0.049). Similarly, MSA patients with constipation showed a trend toward lower plasma cf-mtDNA copy numbers than those without this symptom (*P* = 0.076). MSA patients with cognitive impairment had significantly higher cf-nDNA levels than those without cognitive impairment (*P* = 0.008). Notably, there was a trend toward higher plasma cf-nDNA levels in PD patients with anxiety or depression compared to PD patients without anxiety or depression (*P* = 0.065, *P* = 0.061).

**TABLE 4:** Comparisons of cfDNA biomarkers between PD and MSA subgroups.

Plasma measures	PD		*P*-value	MSA		*P*-value
	**Without cognitive impairment (*n* = 37)**	**With cognitive impairment (*n* = 25)**		**Without cognitive impairment (*n* = 18)**	**With cognitive impairment (*n* = 15)**	
cf-nDNA level	7.985 ± 2.743	9.095 ± 3.221	0.123	6.852 ± 2.933	9.403 ± 4.272	**0.008**
cf-mtDNA copy number	13.072 ± 10.826	12.462 ± 14.426	0.472	13.530 ± 10.380	11.448 ± 7.303	0.925
cf-mtDNA deletion level	1.077 ± 0.064	1.071 ± 0.041	0.576	1.062 ± 0.077	1.101 ± 0.067	0.226
	**Without depression** **(*n* = 32)**	**With depression** **(*n* = 30)**		**Without depression** **(*n* = 19)**	**With depression** **(*n* = 14)**	
cf-nDNA level	7.901 ± 3.053	9.000 ± 2.820	0.061	7.972 ± 4.448	8.064 ± 2.757	0.392
cf-mtDNA copy number	12.756 ± 11.066	12.900 ± 13.675	0.606	11.431 ± 9.927	14.147 ± 7.748	0.294
cf-mtDNA deletion level	1.079 ± 0.048	1.069 ± 0.063	0.638	1.078 ± 0.072	1.081 ± 0.080	0.975
	**Without anxiety (*n* = 27)**	**With anxiety (*n* = 35)**		**Without anxiety (*n* = 11)**	**With anxiety (*n* = 22)**	
cf-nDNA level	7.557 ± 2.286	9.108 ± 3.280	0.065	8.564 ± 5.662	7.735 ± 2.483	0.291
cf-mtDNA copy number	14.280 ± 11.832	11.704 ± 12.694	0.375	13.570 ± 12.863	12.090 ± 6.702	0.810
cf-mtDNA deletion level	1.076 ± 0.058	1.073 ± 0.054	0.986	1.062 ± 0.069	1.088 ± 0.077	0.549
	**Without RBD (*n* = 34)**	**With RBD (*n* = 28)**		**Without RBD (*n* = 5)**	**With RBD (*n* = 28)**	
cf-nDNA level	8.506 ± 3.054	8.344 ± 2.920	0.779	7.335 ± 2.609	8.132 ± 3.968	0.874
cf-mtDNA copy number	13.861 ± 11.342	11.569 ± 13.463	0.351	11.243 ± 8.107	12.823 ± 9.310	0.940
cf-mtDNA deletion level	1.069 ± 0.060	1.081 ± 0.049	0.631	1.067 ± 0.061	1.082 ± 0.077	0.513
	**Without constipation (*n* = 21)**	**With constipation (*n* = 40)**		**Without constipation (*n* = 5)**	**With constipation (*n* = 28)**	
cf-nDNA level	7.803 ± 2.943	8.749 ± 3.006	0.362	7.727 ± 2.905	8.062 ± 3.948	0.746
cf-mtDNA copy number	17.097 ± 11.896	10.717 ± 12.195	**0.049**	18.947 ± 6.522	11.447 ± 9.037	0.076
cf-mtDNA deletion level	1.086 ± 0.065	1.069 ± 0.050	0.292	1.070 ± 0.033	1.081 ± 0.080	0.948
	**Without olfaction loss (*n* = 29)**	**With olfaction loss (*n* = 33)**		**Without olfaction loss (*n* = 28)**	**With olfaction loss (*n* = 5)**	
cf-nDNA level	8.367 ± 3.242	8.491 ± 2.760	0.821	7.797 ± 3.877	9.214 ± 3.192	0.408
cf-mtDNA copy number	12.879 ± 12.558	12.779 ± 12.252	0.726	12.926 ± 9.511	10.667 ± 6.119	0.804
cf-mtDNA deletion level	1.073 ± 0.053	1.076 ± 0.058	0.508	1.084 ± 0.077	1.058 ± 0.055	0.642

Bold values: *P*-value < 0.05 after adjusted for age, sex, BMI and education years. Data are expressed as the mean ± SD. Definition of abnormal: for cognitive impairment, a cut-off of 26 on the MMSE score; for depression, a cut-off of 8 on the HAMD score; for anxiety, a cut-off of 7 on the HAMA score; for RBD, a cut-off of 19 on the RBDQ-HK score; for olfactory loss, a cut-off of 22 on the AHRS score; constipation was screened by the part 21 of NMSS. RBD, rapid eye movement sleep behavior disorder; cf-mtDNA, circulating cell-free mitochondrial DNA; cf-nDNA, circulating cell-free nuclear DNA; PD, Parkinson’s disease; MSA, multiple system atrophy.

## Discussion

This study offers the first comprehensive assessment of plasma cf-nDNA levels, cf-mtDNA copy numbers, and cf-mtDNA deletion levels in patients with PD and MSA. Our cross-sectional study found no significant differences in plasma cf-nDNA levels, cf-mtDNA copy numbers, and cf-mtDNA deletion levels among the PD, MSA, and NC groups. Additionally, there was no correlations between these cfDNA biomarkers and the risk of developing PD or MSA. Notably, in PD patients, there was a significantly positive correlation between plasma cf-nDNA levels and HAM-A scores. In MSA patients, cf-nDNA levels were positively correlated with ICARS scores and negatively correlated with MoCA scores. Additionally, we found that cf-mtDNA copy numbers were significantly lower in PD patients with constipation compared to those without. MSA patients with cognitive impairment had significantly higher cf-nDNA levels compared to those without.

Plasma cfDNA is derived from DNA released from dying cells, with a half-life of about 5–150 min. This “global snapshot” capability makes it an ideal molecular marker for many diseases, especially neurodegenerative diseases where tissue biopsy is not possible (Song et al., 2022). Many neurological disorders are not characterized by alterations in DNA sequences, and therefore, except for epigenetics, the application of cfDNA from nuclear genes in neurodegenerative disorders has been mostly based on the quantification of cfDNA (Supplementary Table 1). Our study found no significant differences in cf-nDNA levels among the PD, MSA, and NC groups. This suggests that plasma cf-nDNA levels may not serve as a reliable biomarker for diagnosing PD and MSA. Further analysis showed no significant association between plasma cf-nDNA levels and the risk of developing PD or MSA. These findings are consistent with previous reports on cf-nDNA concentrations in PD patients. Wojtkowska et al. (2024) investigated serum cf-nDNA levels in 30 PD patients and 15 NC by detecting the nuclear gene *KRAS*, revealing a non-significant elevation in cf-nDNA levels among PD patients after adjusting for age. In contrast, Scalzo et al. (2009) observed a significant reduction in cf-nDNA levels in PD patients compared to controls, based on detecting the nuclear gene β*-globin* in 42 PD patients and 20 controls. However, two cross-sectional studies from Chen M. et al. (2017) and Chen Y. S. et al. (2017) reported a significant increase in cf-nDNA levels in PD patients. Our study improves on these previous studies. First, they did not consider confounding factors like age, sex, BMI, and years of education when analyzing differences in cf-nDNA levels. Second, low statistical power due to small sample sizes was a significant limitation in most of these studies, potentially leading to inconsistent results. In addition, for quantification of cf-nDNA concentrations, some studies have used simple fluorescence quantification as well as qPCR assays based on the SYBR Green method. Considering our wider subject population, more accurate TaqMan probe qPCR method, and rigorous statistical correction, we believe the presented data in this study will inspire future follow-up studies.

Mitochondrial dysfunction is widely believed to play a crucial role in PD and MSA (Nakamoto et al., 2018; Monzio et al., 2020; Asghar et al., 2022). In 1979, studies showed that the toxin MPTP, which inhibits mitochondrial respiratory complex I, can induce substantia nigra cell loss and Parkinson’s disease (Davis et al., 1979). Additionally, patients with mutations in mitochondrial polymerase γ and other mitochondrial disorders exhibit nigrostriatal neurodegeneration, Lewy body pathology, and clinical features of Parkinson’s disease (Gonzalez-Hunt and Sanders, 2021). Pathological changes in mtDNA gradually accumulate in the brains of patients. Various studies have implicated mtDNA related processes as possible alternative avenues of mitochondrial dysfunction in PD risk (Bender et al., 2006; Lin et al., 2012; Dölle et al., 2016). Therefore, The pathophysiological role of cfDNA, particularly cf-mtDNA, in neurodegeneration is increasingly evident. Released from stressed or damaged cells, cfDNA serves as a potent danger-associated molecular pattern (DAMP) that activates pattern recognition receptors (e.g., TLRs, cGAS/STING) in brain-resident cells such as microglia, astrocytes, and neurons. This activation initiates inflammatory signaling cascades—especially via the NF-κB pathway—that lead to the release of pro-inflammatory cytokines (e.g., IL-1β, IL-6), which intensify neuroinflammation and neuronal injury. Additionally, cf-mtDNA contributes to oxidative stress, compounding cellular damage and further promoting inflammation. In the aging brain, where mitochondrial dysfunction and oxidative stress are already elevated, cfDNA levels tend to rise, perpetuating chronic inflammation that drives neurodegenerative progression (Kunze et al., 2023; Legati and Ghezzi, 2023). Several studies have linked CSF-derived cf-mtDNA to PD. Pyle et al. (2015) examined CSF from PD patients and found significantly reduced levels of cf-mtDNA compared to healthy controls. Lowes et al. (2020) found that the reduction in CSF cf-mtDNA copy number may be related to the initiation, type, and duration of treatment. Similar studies have been conducted in other neurodegenerative diseases, including Alzheimer’s disease (Podlesniy et al., 2020) and multiple sclerosis (Varhaug et al., 2017; Fissolo et al., 2019). This suggests that assessing mitochondrial molecules involved in neurodegenerative processes may provide a sensitive indicator of mitochondrial dysfunction in neurodegenerative diseases.

cfDNA can cross the blood-brain barrier and enter the peripheral circulation, thus measuring disease-related changes in cf-mtDNA copy numbers and deletion levels in blood samples is appealing. As shown in Supplementary Table 1, there has been a significant increase in research on cf-mtDNA in serum or plasma as potential biomarkers for neurodegenerative diseases, indicating the great potential of blood cf-mtDNA as a non-invasive biomarker for these diseases. This study is the first to find no statistically significant differences in plasma cf-mtDNA copy numbers and cf-mtDNA deletion levels between PD and MSA patients and healthy controls. Similarly, Borsche et al. (2020) reported no differences in serum cf-mtDNA levels between idiopathic PD patients and healthy controls. However, affected heterozygous and double allele *PRKN/PINK1* mutation carriers have higher serum cf-mtDNA levels than idiopathic PD patients and healthy controls (Borsche et al., 2020). Similar results were obtained in two studies focusing on CSF from the same team. They found that high cf-mtDNA levels in CSF occurred only in *LRRK2* mutation carriers with PD, not in idiopathic PD patients (Podlesniy et al., 2016; Puigròs et al., 2022). These findings were in agreement with the work published by Wojtkowska et al. (2024), who demonstrated that after correcting for the confounding factor of age, there were no significant differences in serum or CSF cf-mtDNA levels in PD patients compared to NC. While various forms of cell death occur in neurons or glial cells within the brain of patients, leukocytes are the primary contributors to almost 50–80% of cfDNA derived from plasma. Neutrophil-derived cfDNA makes up the majority, while neuron-derived cfDNA represents only 1–2% of the plasma DNA pool (Sun et al., 2015; Moss et al., 2018; Loyfer et al., 2023). This fraction of cfDNA in PD and MSA patients is likely released from peripheral blood neutrophils, similar to what is observed in cancer patients and healthy individuals undergoing acute exercise (Fridlich et al., 2023; Mattox et al., 2023). Therefore, the results of this experiment may reflect the mitochondrial function of the patient’s general immune cells rather than brain-specific mitochondrial dysfunction. Although many studies on neurodegenerative diseases have reported changes in cf-mtDNA copy numbers and deletion levels, differences in assay processes across laboratories often result in non-comparable data. These differences include variations in target genes, biological sample types, blood collection timing, centrifugation conditions, DNA extraction methods, and qPCR protocols (Trumpff et al., 2021; Park et al., 2022). Inconsistent results have been observed between different neurodegenerative diseases and even in the same disease, such as Parkinson’s disease. This suggests that the relationship between cf-mtDNA and neurodegenerative diseases may be more complex, reflecting the diversity and multifaceted etiology of each disease. Additionally, sample heterogeneity and inaccuracies in cf-mtDNA assay methods may partly explain the conflicting results frequently reported in the literature. Therefore, establishing standardized blood processing methods and analytical workflows is essential, along with the systematic characterization of both cf-mtDNA and cf-nDNA, potentially utilizing samples from large clinical cohorts (Bronkhorst et al., 2022). In this study, a two-step centrifugation method was used, with no significant impact on nuclear DNA. However, at a force of 16,000 *g*, larger structures like platelets precipitate, leading to a decline in mtDNA concentration in the plasma supernatant and the loss of around 75% of mtDNA. Consequently, it needs to be clarified that the analyzed cf-mtDNA was related to small extracellular vesicles, exosomes, and protein complexes under such centrifugal conditions (Pisareva et al., 2023). Future studies need to consider the limitations of previous research and the challenges of analyzing mtDNA to improve study designs.

In synucleinopathies, α-synuclein released from neurons or oligodendrocytes activates microglia, initiating an inflammatory response in the central nervous system (Yuan et al., 2024). This inflammatory and immune response is observed not only in the central nervous system, but also in the peripheral blood (Passaro et al., 2021; Jun and Kim, 2023). Neutrophils release neutrophil extracellular traps (NETs) when activated by various endogenous and exogenous inflammatory factors, further contributing to inflammation. NETs can directly release DNA fragments into the microenvironment, creating a positive feedback loop that perpetuates chronic inflammation (Pisetsky, 2012). Furthermore, two studies have reported an association between α-synuclein and NETs formation (Azevedo et al., 2012; Peelaerts et al., 2023). Notably, we observed PD patients with anxiety or depression tended to have higher plasma cf-nDNA levels compared to those without these conditions. Previous studies (Eraly et al., 2014; Milaneschi et al., 2021; Zeng et al., 2024) have found that associations of C-reactive protein, lymphocyte to monocyte ratio and neutrophil to lymphocyte ratio with the risks of any psychiatric disorder, depression, anxiety, and stress-related disorders. Possible explanations include blood-brain barrier disruption, microglial activation, neurotransmission disorders, and interactions between inflammation and neuropathology (Zeng et al., 2024). This aligns with our findings that cf-nDNA levels in PD patients are positively correlated with HAM-A scores. In addition, our results indicate that cf-nDNA levels were significantly elevated in MSA patients exhibiting cognitive impairment than those with normal cognition, and a significantly negative correlation was identified between MoCA scores and cf-nDNA levels in MSA patients. This is consistent with previous findings. Nidadavolu et al. (2022) analyzed serum cf-nDNA from 631 community-dwelling individuals, finding that high baseline cf-nDNA levels were associated with lower overall cognitive functioning, increased risk of dementia, and faster cognitive decline over 8 years. We also found a significantly positive correlation between plasma cf-nDNA levels and ICARS scores in MSA patients. Although the mechanisms linking cfDNA levels with cognitive loss and ataxia symptoms remain unclear, this is not surprising given MSA’s inflammatory nature and cfDNA’s role as a biomarker of systemic inflammation (Leńska-Mieciek et al., 2023). Chronic neuroinflammation in MSA patients’ brains leads to neuronal damage and blood-brain barrier disruption, releasing DNA into peripheral circulation. These cfDNAs themselves can also act as DAMPs to induce immune responses, triggering further inflammation and peripheral cell death (Chen et al., 2015; Kunze et al., 2023). Although we could not determine a causal relationship between the clinical symptoms and plasma cf-nDNA levels, this result paves the way for further studies on the correlation between plasma cf-nDNA levels and MSA severity. Another notable finding is that, although both PD and MSA are α-synucleinopathies, the correlations mentioned above do not show a consistent trend between these diseases. This may suggest that, despite sharing common pathological mechanisms, PD and MSA exhibit different peripheral inflammation and immune characteristics (Folke et al., 2019; Yuan et al., 2024), which could lead to variations in the interactions between cf-nDNAs and the inflammatory responses. It should also be noted that, beyond concentration, cfDNA fragment size and the specific sequences of cfDNA are related to the inflammatory responses (Storci et al., 2018; Deng et al., 2022). This highlights the need for further research to better understand the specificities and commonalities of these diseases.

Constipation is one of the most common functional gastrointestinal disorders in patients with PD and MSA. Evidence suggests that both PD and MSA patients exhibit disruption of intestinal barrier integrity, endotoxin-mediated intestinal inflammation, and pro-inflammatory microbiota. This intestinal-sourced inflammatory cascade may lead to local and systemic inflammation, elevated pro-inflammatory cytokines, and oxidative stress (Engen et al., 2017; Tan et al., 2021). At the same time, changes in microbial metabolites caused by intestinal microbiota imbalance in PD patients can also affect mitochondrial dysfunction (Liang et al., 2021). This is consistent with our results. Our study showed that PD and MSA patients with constipation had lower cf-mtDNA copy numbers compared to those without. Considering the high energy demands of neural, muscular, and inflammatory cells involved in gastrointestinal tract function (Camilleri et al., 2009), and the fact that low mtDNA copy number is associated with high inflammatory markers (Wu et al., 2017; Kang et al., 2021), it is plausible that patients with constipation present with reduced cf-mtDNA copy numbers. Another piece of evidence for this phenomenon is that previous research has linked constipation to reduced mtDNA copy numbers in individuals with autism spectrum disorder and intellectual disability compared to NC (Valiente-Pallejà et al., 2018). Future studies should further explore the complex interactions between intestinal inflammation and mitochondrial dysfunction to reveal new therapeutic targets.

Limitations of this study need to be noted. Firstly, the sample size was relatively small and the study is single-centered. Findings in this study need to be validated in larger, more diverse sample groups and multiple centers. Secondly, demographic data show a significant difference in the history of hypertension, coronary heart disease and cerebrovascular disease among the three groups. Whether this difference leads to changes in plasma cf-nDNA or cf-mtDNA is unknown. To address this, we conducted additional statistical analyses and the results are shown in Supplementary Table 2. Overall, there were no significant differences in plasma cf-nDNA or cf-mtDNA levels between subjects with hypertension, coronary heart disease, cerebrovascular disease, and those without these comorbidities. This suggests that these comorbidities may not significantly impact plasma cf-nDNA or cf-mtDNA levels. Considering that both PD and MSA are age-related, it is likely that all subjects will have some degree of comorbidities. Future research should focus on the impact of various comorbidities on cfDNA levels in the elderly and rigorously control for these factors statistically to minimize their impact. Thirdly, since simultaneous release of nuclear and mitochondrial genomes may indicate cell death or injury, we opted to assess changes in mtDNA copy numbers using real-time quantitative PCR, quantifying the ratio of mitochondrial to nuclear genomes. This method produces relative measurements compared to nuclear DNA copy numbers in the sample rather than absolute mtDNA copy number values, it complicates cross-study comparisons and objective clinical utility assessments. Future studies might consider using digital droplet PCR for absolute mtDNA copy number assessment and standardized pre-analytical cf-mtDNA procedures. Finally, plasma cf-nDNA concentrations are exceedingly low due to limited plasma inputs and the fragmented double-stranded structure of cfDNA. Therefore, the elevated Cq values observed in quantitative PCR analyses may have compromised the study’s reliability. Future investigations could mitigate this issue by developing reference primers targeting either short or long interspersed nuclear elements.

In future neurodegenerative disease research, plasma cfDNA markers alone may have limited utility but could be combined with other biomarkers to enhance specificity. For instance, cfDNA could be combined with DNA methylation indicators. DNA methylation profiles can help trace the tissue origin of cfDNA from damaged cells, as cfDNA fragments retain tissue-specific methylation patterns. Additionally, certain differentially methylated sites are strongly linked to neurodegenerative diseases and exhibit high specificity. Beyond cfDNA methylation, cfDNA markers could be integrated with symptomatic markers, imaging markers, biochemical markers, and genetic markers to significantly enhance diagnostic sensitivity and specificity. A promising future approach involves using AI models to integrate multimodal data—demographics, medical history, lab results, medication profiles, neuropsychological tests, functional assessments, and multimodal neuroimaging—to diagnose and identify α-synucleinopathies.

In conclusion, our study suggests that plasma cf-nDNA level, cf-mtDNA copy number, and cf-mtDNA deletion level may not be distinct indicators for identifying PD and MSA patients. Moreover, significant associations between these three cfDNA biomarkers and the risk of developing PD and MSA are lacking. Nevertheless, their notable correlations with clinical features suggest that these biomarkers can still offer valuable insights into disease burden and progression. These findings underscore the necessity for further exploration of these relationships and the clinical applications of these biomarkers in PD and MSA diagnosis and management.

## Data Availability

The raw data supporting the conclusions of this article will be made available by the authors, without undue reservation.
