# Supplementary material for: Probing the diagnostic values of plasma cf-nDNA and cf-mtDNA for Parkinson’s disease and multiple system atrophy
Source: Front Neurosci. 2024 Dec 2;18:1488820. doi: 10.3389/fnins.2024.1488820 (PMC11647036; doi:10.3389/fnins.2024.1488820)
Supplement: Supplementary file 1 [file Table_1.doc]

**Supplementary Table 1**

An overview of studies on cf-nDNA and cf-mtDNA in plasma and serum associated with neurodegenerative disorders.

| Neurological disorders | Study | PMID number | Publication year | Biofluid sample | Biomarker | Methodology | Study group | Results |
| --- | --- | --- | --- | --- | --- | --- | --- | --- |
| AD | Pai et al. | 30032422 | 2018 | Plasma | cf-nDNA | qPCR | AD (n = 27) NC (n = 9) | The cf-nDNA levels were significantly increased in the AD patients compared with NC (*P* < 0.01). |
| AD | Silzer et al. | 30861027 | 2019 | Plasma | cf-mtDNA | qPCR | T2D only (n = 17) CI only (n = 5) Both T2D and CI (n = 6) NC (n = 18) | Diabetic individuals had significantly higher cf-mtDNA levels compared to non-diabetic individuals (*P* = 0.026). There was no significant difference in cf-mtDNA levels between CI individuals and cognitively intact individuals (*P* = 0.067). cf-mtDNA levels appear to be significantly higher in individuals suffering from both diseases (T2D and CI) when compared to NC (*P* = 0.024). |
| PD | Scalzo et al. | 19444939 | 2009 | plasma | cf-nDNA | qPCR | PD (n = 42) NC (n = 20) | Significant reduction in cf-nDNA levels in PD patients relative to NC (*P* < 0.001). |
| PD | Sharma et al. | 32125208 | 2021 | serum | cf-mtDNA | qPCR | PD males (n = 30)  PD females (n = 17) NC males (n = 20) NC females (n = 20) | Increase in cf-mtDNA serum concentrations was observed in PD females compared to healthy females (*P* < 0.0001), while there was no difference in serum concentrations between PD males and healthy males. |
| PD | Borsche et al. | 33029617 | 2020 | serum | cf-mtDNA | qPCR | PRKN/PINKI biallelic  （mut^+/+^ PD^+^，n = 13） PRKNIPINKI affected heterozygous  （mut^+/–^ PD^+^，n = 17） PRKN/PINKI unaffected heterozygous（mut^+/–^ PD^–^，n = 14） IPD（n = 57） NC (n = 55) | Compared to patients with IPD, pairwise analyses showed increased cf-mtDNA release in PRKN/PINK1 biallelic mutation carriers (*P* = 0.0094) and affected heterozygous mutation carriers (*P* = 0.0002).  Compared to NC, a trend towards elevated cf-mtDNA levels in biallelic PRKN/PINK1 mutation carriers (*P* = 0.0459) and an increase in affected heterozygous individuals (*P* = 0.0019).  Elevated cf-mtDNA levels in affected compared to unaffected PRKN/ PINK1 heterozygotes (*P* = 0.0058). |
| PD | Chen et al. | 28232858 | 2017 | plasma | cf-nDNA and cf-mtDNA | qPCR | PD (n = 29) NC (n = 26) | Compared to the NC, PD patients exhibited higher cf-nDNA level (*P* < 0.001) and cf-mtDNA level (*P* = 0.023). |
| PD | Chen et al. | 28174514 | 2017 | plasma | cf-nDNA and cf-mtDNA | qPCR | EOPD (n = 24)  NC (n = 33) | The plasma cf-nDNA levels were significantly increased (*P* = 0.026) in the EOPD patients; the plasma cf-mtDNA levels showed no significant difference (*P* = 0.406). |
| PD | Chen et al. | 33680283 | 2021 | plasma | cf-nDNA and cf-mtDNA | qPCR | PDN (n = 25)  PDMCI (n = 25) PDD (n = 38) NC (n = 47) | Significantly higher plasma cf-nDNA levels in the PDD group compared with the NC, PDN, and PDMCI groups (all *P* < 0.001). No significant plasma cf-mtDNA difference was detected among all subgroups. |
| PD | Wojtkowska et al. | 38474065 | 2024 | serum | cf-nDNA and cf-mtDNA | ddPCR | PD (n = 30) NC (n = 15) | Compared with NC, there is no significant difference in serum cf-nDNA (*P* =0.338) and cf-mtDNA levels (*P* = 0.063) in PD patients. |
| FRDA | Swarup et al. | 21329459 | 2011 | Plasma | cf-nDNA | Pico Green dsDNA quantitation assay | SCA12 (n = 25)  SCA2 (n = 10)  FRDA (n = 15) NC (n = 20) | The plasma cf-nDNA levels of patients with FRDA, SCA2, and SCA12 were significantly higher than those of NC (all *P* < 0.001). |
| FRDA | Dantham et al. | 27206881 | 2016 | Plasma | cf-nDNA and cf-mtDNA | Multiplex qPCR | FRDA (n = 21) NC (n = 21) | the levels of cf-nDNA found to be increased (*P* < 0.005), whereas cf-mtDNA levels were reduced significantly in the plasma of FRDA patients as compared to NC (*P* < 0.001). |
| MS | Liggett et al. | 20064646 | 2010 | Plasma | cf-nDNA | fluorescence | RRMS(r) (N = 30) RRMS(e) (N = 29) NC (N = 30) | The concentration of cf-nDNA is significantly higher in both RRMS(r) (*P* < 0.0001) and RRMS(e) (*P* < 0.005) groups as compared to NC. The concentration of cf-nDNA in RRMS(r) is significantly higher than in RRMS(e) (*P* < 0.02). |
| MS | Nasi et al. | 31726376 | 2020 | Plasma | cf-mtDNA | qPCR | SP (N = 34) PP (N = 26)  NC (N = 30) | cf-mtDNA is higher in SP patients compared to PP patients (*P* = 0.004) |

Abbreviations: cf-mtDNA: circulating cell-free mitochondrial DNA; cf-nDNA: circulating cell-free nuclear DNA; qPCR: quantitative Polymerase Chain Reaction; ddPCR: droplet digital polymerase chain reaction; AD: Alzheimer's disease; CI: Cognitive Impairment; PD: Parkinson’s disease; NC: Normal control; MS: Multiple sclerosis; T2D: Type 2 Diabetes; EOPD: Early Onset Parkinson’s disease; PDN: Parkinson’s disease patients with normal cognition; PDMCI: Parkinson’s disease patients with mild cognitive impairment; PDD: Parkinson’s disease patients with dementia; IPD: Idiopathic Parkinson's disease; SCA: spinocerebellar ataxia; FRDA: Friedreich’s ataxia; SP: secondary progressive patients; PP: primary progressive patients; RRMS: Relapsing-remitting multiple sclerosis; RRMS(r): Relapsing-remitting multiple sclerosis in remission; RRMS(e): Relapsing-remitting multiple sclerosis in exacerbation.
