# Supplementary material for: Probing the diagnostic values of plasma cf-nDNA and cf-mtDNA for Parkinson’s disease and multiple system atrophy
Source: Front Neurosci. 2024 Dec 2;18:1488820. doi: 10.3389/fnins.2024.1488820 (PMC11647036; doi:10.3389/fnins.2024.1488820)
Supplement: Supplementary file 2 [file Table_2.doc]

**Supplementary Table 2**

Comparison of the levels of cfDNA biomarkers between subject with and without HP, CHD, CVD.

| Group | Plasma measures | HP^a^ | CHD^b^ | CVD^c^ |
| --- | --- | --- | --- | --- |
|  |  | Yes vs. No | Yes vs. No | Yes vs. No |
| ALL |  | N_yes_ = 68, N_no_ = 103 | N_yes_ = 16, N_no_ = 155 | N_yes_ = 21, N_no_ = 150 |
|  | cf-nDNA level | 0.422 | 0.349 | 0.618 |
|  | cf-mtDNA copy number | 0.124 | 0.261 | 0.178 |
|  | cf-mtDNA deletion level | 0.443 | 0.089 | 0.846 |
| NC |  | N_yes_ = 31, N_no_ = 45 | N_yes_ = 2, N_no_ = 74 | N_yes_ = 0, N_no_ = 76 |
|  | cf-nDNA level | 0.658 | 0.781 | - |
|  | cf-mtDNA copy number | 0.203 | 0.329 | - |
|  | cf-mtDNA deletion level | 0.980 | 0.903 | - |
| PD |  | N_yes_ = 30, N_no_ = 32 | N_yes_ = 13, N_no_ =49 | N_yes_ = 15, N_no_ = 47 |
|  | cf-nDNA level | 0.482 | 0.201 | 0.474 |
|  | cf-mtDNA copy number | 0.097 | 0.281 | 0.510 |
|  | cf-mtDNA deletion level | 0.636 | **0.049** | 0.446 |
| MSA |  | N_yes_ = 7, N_no_ = 26 | N_yes_ = 1, N_no_ = 32 | N_yes_ = 6, N_no_ = 27 |
|  | cf-nDNA level | 0.729 | 0.107 | 0.191 |
|  | cf-mtDNA copy number | 0.853 | 0.588 | 0.185 |
|  | cf-mtDNA deletion level | **0.037** | 0.570 | 0.341 |

Bold values: *P* value < 0.05.

Abbreviations: HP: Hypertension; CHD: Coronary heart disease; CVD: Cerebrovascular disease; NC: Normal control; PD: Parkinson's disease; MSA: Multiple system atrophy; cf-mtDNA: circulating cell-free mitochondrial DNA; cf-nDNA: circulating cell-free nuclear DNA; -: Not available.

a: adjusting for age, sex, BMI, education years, CHD and CVD for all, PD and MSA groups; adjusting for age, sex, BMI, education years and CHD for NC group.

b: adjusting for age, sex, BMI, education years, HP and CVD for all, PD and MSA groups; adjusting for age, sex, BMI, education years and HP for NC group.

c: adjusting for age, sex, BMI, education years, HP and CHD for all, PD and MSA groups.
